# Cerium Dioxide Nanoparticles as Smart Carriers for Self-Healing Coatings

**DOI:** 10.3390/nano10040791

**Published:** 2020-04-20

**Authors:** Sehrish Habib, Eman Fayyad, Muddasir Nawaz, Adnan Khan, Rana A. Shakoor, Ramazan Kahraman, Aboubakr Abdullah

**Affiliations:** 1 Center for Advanced Materials (CAM), Qatar University, Doha 2713, Qatar; sehrish.habib@qu.edu.qa (S.H.); emfayad@qu.edu.qa (E.F.); m.nawaz@qu.edu.qa (M.N.); ak1704740@qu.edu.qa (A.K.); bakr@qu.edu.qa (A.A.); 2 Department of Chemical Engineering, Qatar University, Doha 2713, Qatar; ramazank@qu.edu.qa

**Keywords:** nanocomposite coating, self-healing, corrosion inhibitor, electrochemical impedance spectroscopy, nanoparticles

## Abstract

The utilization of self-healing cerium dioxide nanoparticles (CeO_2_), modified with organic corrosion inhibitors (dodecylamine (DDA) and n-methylthiourea (NMTU)), in epoxy coating is an efficient strategy for enhancing the protection of the epoxy coating and increasing its lifetime. Fourier transform infrared (FTIR) spectroscopy analysis was used to confirm the loading and presence of inhibitors in the nanoparticles. Thermal gravimetric analysis (TGA) measurement studies revealed the amount of 25% and 29.75% *w/w* for NMTU and DDA in the nanoparticles, respectively. The pH sensitive and self-release behavior of modified CeO_2_ nanoparticles is confirmed through UV-vis spectroscopy and Zeta potential. It was observed, through scanning electron microscopy (SEM), that a protective layer had been formed on the defect site separating the steel surface from the external environment and healed the artificially created scratch. This protective film played a vital role in the corrosion inhibition of steel by preventing the aggressiveness of Cl^−^ in the solution. Electrochemical impedance spectroscopy (EIS) measurements exhibited the exceptional corrosion inhibition efficiency, reaching 99.8% and 95.7% for the modified coating with DDA and NMTU, respectively, after five days of immersion time.

## 1. Introduction

Corrosion results in significant materials loss and equipment failure and is scientific challenge worldwide. According to one survey, about a quarter to a third of the total downtime in industries is due to the deleterious effects of corrosion [1]. It is, therefore, essential to prevent corrosion to ensure the reliability of the assets [2]. Usually, the protection of piping steel against corrosion is achieved by applying thick organic coatings [3,4,5]. These coatings provide a decent barrier protection against aging effects, mechanical scratches, erosion, and other damages [6]. The protection of damaged piping parts requires steel repair and re-coating, which is a costly process. To minimize the impact of damages and subsequent corrosion activity of the steel, it is essential to act fast and efficiently, preferably in an autonomous way. Modern trends indicate that smart, functional coatings, containing corrosion inhibition species are attractive for prolonged lifetime of materials [7,8].

Different types of anti-corrosive coatings are reported in the literature [9,10]. Organic and inorganic corrosion inhibitors loaded in different delivery systems like halloysite nanotubes [11], titanium nanotubes [12], and mesoporous silica [13] have been used in epoxy coatings. Corrosion inhibitors have a direct effect on the entire metallic surface when released in a sufficient amount upon the initiation of the corrosion process [14]. Organic inhibitors, such as amines, aldehydes, alkaloids, and nitro compounds, have been analyzed and used as corrosion inhibitors in epoxy coatings [15]. These organic inhibitors form a protective passive layer when released, either by the reaction between the metal and the environment, or by the interaction of the surface charges with the dipole charges on the metallic surface [16]. Based on their reaction at the metallic surface, the organic inhibitor can be anodic or cathodic. Anodic inhibitors such as chromates, phosphates, and tungstate increase the anodic polarization and hence increase the cathodic corrosion potential, whereas the cathodic inhibitors reduce the corrosion rate by slowing down the reduction reaction [17]. The effectiveness of these organic inhibitors depends upon their size, carbon-chain length, aromaticity, conjugation, and nature of bonding atoms. These coatings, modified with an organic corrosion inhibitor delivery system, can prevent corrosion at an early stage, minimizing corrosion onset and corrosion propagation [18]. The key purpose of the corrosion-resistant coatings is to shield the steel substrate from the external environment. Thus, deterring of corrosion activity on steel is considered self-healing. The self-healing process can be described in three steps. (1) The reservoir carrying healing agent is ruptured when the scratch is made; (2) release of corrosion inhibitor to cover the crack; (3) formation of a new layer of the deposited inhibitor, or healing of the crack due to reaction with the external environment. Thus, this process automatically heals the damage and extends the life of protective coatings [19]. Consequently, they are a promising solution for a longer durability of coated piping steel and decreased operation expenses contributing to economic savings, materials reliability, and safety. Liu et al. [20] successfully synthesized water-based epoxy coatings containing loaded CeO_2_ nanocontainers with BTA, and deposited a polyelectrolyte multilayer on it by the layer-by-layer technique. Electrochemical impedance spectroscopy (EIS) and Scanning Kelvin probe (SKP) studies revealed promising results relating to the inhibition of corrosion activity on steel surfaces. R.Z et al. [21] studied the effect of ceria and zirconia nanoparticles on the corrosion inhibition behavior of unmodified cerium and modified silane coatings in 3 and 5 wt. % NaCl solution on electrogalvanized steel substrate. Tavandashti et al. [22] synthesized pH sensitive Ce(III) -PANI complex and studied the pH triggered the release of Ce (III) from a complex, which resulted in decent corrosion inhibition performance. Mekeridis et al. [23] investigated the release behavior of inhibitor 2-mercaptobenzothiazole (2-MB) and 8-hydroxyquinoline (8-HQ) from modified cerium titanium oxide nano container in corrosive media via the EIS technique on aluminum alloys (AA2024-T3).

**Figure 1:**
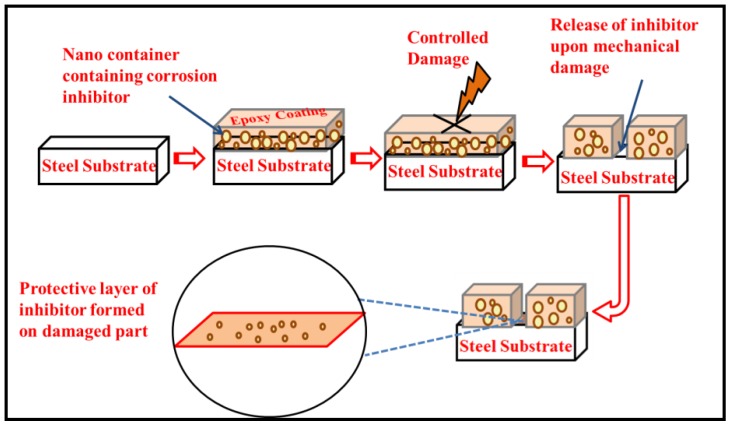
Proposed methodology and protective mechanism of epoxy reinforced nano composite coating.

CeO_2_ nanoparticles are considered to be very effective as carriers or reservoirs for various types of corrosion inhibitors [21]. In the current study, we evaluated the loading ability of two different organic inhibitors (dodecylamine (DDA) and n-methyl thiourea (NMTU)) that were loaded separately in the cerium dioxide (CeO_2_) nanoparticles. The full investigation is done for the morphology, elemental analysis, and phases of the unmodified and modified CeO_2_, with the inhibitors (CeO_2_/DDA and CeO_2_/NMTU) using transmission electron microscopy (TEM), energy dispersive X-ray spectroscopy (EDS) and X-ray diffraction (XRD), respectively. Fourier transform infrared (FTIR) and thermal gravimetric analysis (TGA) were utilized to elucidate the structure and the thermal properties, respectively, of the CeO_2_, CeO_2_/DDA, and CeO_2_/NMTU before and after embedding them in the epoxy coating. Furthermore, the self-release capability of the inhibitors was traced as a function of pH at different times (24, 48, 72, and 96 h) using UV-vis spectroscopy. Finally, the self-healing performance of the scratched nanocomposite epoxy coating that was immersed in a 3.5 wt.% NaCl solution was physically and electrochemically investigated using scanning electron microscope (SEM) and electrochemical impedance spectroscopy (EIS). To the best of our knowledge, no studies were made on the effectiveness of the self-healing and the corrosion inhibition efficiency of these two inhibitors loaded into cerium oxide nanoparticles. The proposed self-healing and corrosion inhibition mechanism of epoxy reinforced nanocomposite coatings is shown in Figure 1.

## 2. Experimental

### 2.1. Materials and Chemicals

Cerium dioxide (CeO_2_) nanoparticles, used as nano reservoirs, NMTU and DDA, used as corrosion inhibitors, sodium chloride (NaCl), ethanol, epoxy resin (815C), and its curing agent (EPIKURE Curing Agent 3282) were all purchased from Sigma Aldrich, Darmstadt, Germany. Low carbon steel specimens (30 × 30 × 1.0 mm^3^), used as substrates, were purchased from a local source. Silicon carbide (SiC) abrasive papers were purchased from Hebei Yineng Pipeline Group Co., Ltd., China.

### 2.2. Modification of CeO_2_ Nanoparticles

The modification process involved the preparation of a saturated solution of inhibitor in the respective solvent. For NMTU, water was used as a solvent and for DDA, ethanol was used. About 6.01 g of each corrosion inhibitors was added into 50 mL of the respective solvent. Then, 3.05 g of CeO_2_ nanoparticles were added in the solution. The beaker was sealed to avoid splashing and evaporation of the solution. After being stirred for 18–22 h, the solution was placed in a vacuum for about 2 h to draw out air and to ensure complete loading of corrosion inhibitor in CeO_2_ nanoparticles. The product was then collected by centrifugation, followed by drying in an oven at 60 °C overnight.

### 2.3. Preparation of Epoxy-Based Nanocomposite Smart Coatings

To prepare the epoxy-based nanocomposite smart coatings, the following procedure was adopted. Five grams of epoxy was mixed with 1.25 g of its curing agent. Modified and unmodified CeO_2_ nanoparticles were incorporated into the epoxy mixture at a concentration of 5 wt.% and sonicated at degasser mode for 5–7 min to remove air bubbles from the epoxy mixture and to attain uniform dispersion of CeO_2_ nanoparticles in the mixture. For comparison purposes, a blank epoxy coating and coating with unmodified CeO_2_ nanoparticles (5 wt.%) were also prepared. For simplification, the coatings that are reinforced with unmodified CeO_2_, modified CeO_2_ with DDA, and modified CeO_2_ with NMTU are named as epoxy/CeO_2_, epoxy/CeO_2_/DDA and epoxy/CeO_2_/NMTU, respectively.

### 2.4. Coating Application

The surface of the steel substrate was cleaned and polished beforehand using SiC (120 grit size) abrasive papers. The prepared substrates were then thoroughly rinsed with water and finally cleaned with ethanol before applying coatings. The prepared epoxy-based nanocomposite coatings mixture was then applied on steel substrates using a doctor’s blade technique. Finally, the as-prepared coating samples were left for 15 days curing, until the coating was completely dried and hardened with a final thickness of 50 ± 5 µm, determined by adjusting the gap between the doctor blade and the steel substrate.

### 2.5. Characterization of Modified Nanoparticles

A transmission electron microscope TEM TALOS F200X (FEI, New York, NY, USA) and EDX tool was used to study the microstructure of unmodified and modified CeO_2_ nanoparticles. For this purpose, the sample powder was dispersed in isopropyl alcohol and sonicated for about 5 min. A 20 μL of the dispersed solution was dropped over a 300 mesh copper/lacey carbon grid and dried at room temperature for TEM analysis. The specific surface area (SSA) was calculated by the Brunauer-Emmett-Teller-BET (Surface Area Analyzer, Micromertitcs ASAP 2420, USA,) method. An X-ray diffraction analysis PAN analytical (Empyrean, Royston, UK) X’-pert Pro Cu (Kα) with a scanning rate of 2°/min and scanning angle ranging between 10° ≤ 2θ ≤ 90° was employed to study the structural and phase analysis of samples. Fourier transform infrared (FTIR) spectra were recorded using FTIR Frontier (PerkinElmer, Waltham, MA, USA) spectrometer in range of 4000–500 cm^−1^, to study the confirmation of impregnated particles in CeO_2_ nanoparticles. Thermal stability study for as-received CeO_2_ and CeO_2_ loaded with inhibitor was conducted using TGA/DTA analyzer pyris 4000 (PerkinElmer, USA), ranging from 30 °C to 600 °C at a heating rate of 10 °C/min. In order to study the release behavior of inhibitors from CeO_2_ at different pH values (2, 5, 7, 9, 11) UV-vis spectroscopy was employed using UV-vis spectrophotometer (Biochrome Libra S60 double beam spectrophotometer, United Kingdom). For this purpose, 1 mg of each modified CeO_2_ nanoparticles were added into 3.5 wt.% NaCl solution and absorbance intensities was measured for different time intervals. The zeta potential of unmodified and modified CeO_2_ nanoparticles at various pH was studied at pH (2, 5, 7, 9, and 11) using (Malvern MPT-2 Zetasizer Nano, USA).

### 2.6. Characterization of Smart Coatings

FTIR spectroscopy was used to study the structural analysis of coatings. Thermal stability of the prepared coatings was determined by TGA analysis. SEM (FE-SEM-Nova Nano-450, FEI, New York, NY, USA) was employed to study the self-healing ability of smart coatings. In order to study the corrosion inhibition performance of the synthesized smart coatings in 3.5 wt.% NaCl solution was evaluated using the EIS technique. For this purpose, the coatings were scratched and then electrochemically measured. The electrochemical measurements were carried out using a Gamry 3000 (30K BOOSTER potentiostat/Galvanstate/ZRA, USA), which has a three-electrode system. In this study, the coated sample with an exposed area of 1.0 cm^2^ was used as the working electrode, whereas a graphite rod and Ag/AgCl were used as counter and reference electrodes, respectively. EIS measurements were commenced after attaining the open circuit potential to a steady state value as a function of time. The frequency range for the EIS experiment was within 0.1–100 × 10^3^ Hz and the RMS signal was 10.0 mV. The measured EIS data was analyzed by Gamry (E-Chem 3000 software, USA), and the obtained fitting parameters values were determined by the suitable equivalent circuits. FE-SEM was employed to study the EDX and elemental mapping of the scratched area.

## 3. Results and Discussion

### 3.1. Morphological and Structural Analysis

**Figure 2:**
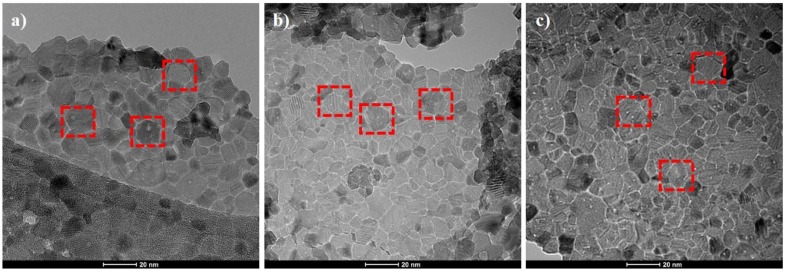
Transmission electron microscopy (TEM) images of (**a**) unmodified CeO_2_ nanoparticles and (**b**,**c**) the modified CeO_2_/DDA and CeO_2_/NMTU, respectively.

Figure 2 shows the TEM images of unmodified and modified CeO_2_ nanoparticles. TEM image shows the size of unmodified cerium oxide nanoparticles ranging between 10–30 nm. The sample possesses a cubic structure with a uniform distribution of CeO_2_ particles that are highlighted with red blocks in Figure 2. The EDX analysis of the CeO_2_ nanoparticles, which is shown in Table 1, confirms the presence of Ce and O elements, as shown in Figure S1 (Supplementary data). The absence of any impurity confirms the purity of CeO_2_. The detected carbon signal in the cerium dioxide sample may be due to the generation of signals from conductive tap used to stick the sample powder on the sample holder. These cubic structures are selected as the carrier for loading of inhibitor molecules (DDA and NMTU). It can also be seen in Figure 2b,c that the morphology of CeO_2_ nanoparticles are still unchanged (highlighted) after modification. Moreover, the EDS analysis of the modified CeO_2_, Table 1, confirmed the presence of carbon and nitrogen elements of the inhibitors confirming the presence of inhibitors on CeO_2_.

The Brunauer-Emmett-Teller (BET) specific surface area (SSA) of the CeO_2_ was determined to be 97.57 m^2^g^−1^, and cumulative pore volume was calculated to be 0.619723 ccg^−1^. The BET measurements of CeO_2_/DDA and CeO_2_/NMTU show that the SSA of the DDA modified CeO_2_ is reduced to be 79.42 m^2^g^−1^, and that of the NMTU modified CeO_2_ is reduced to be 54.32 m^2^g^−1^. In addition, the pore volume is decreased to 0.37396 and 0.32950 ccg^−1^ for DDA and NMTU, respectively, elucidating the loading of inhibitor on CeO_2_ nanoparticles.

**Figure 3:**
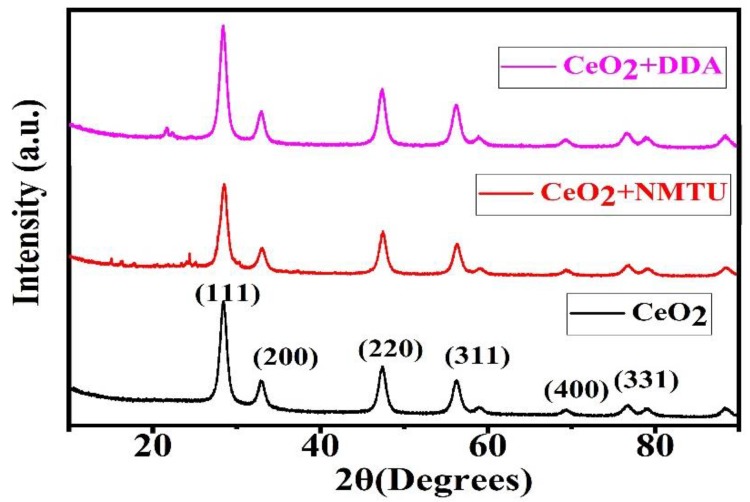
X-ray diffraction (XRD) diffraction spectra of unmodified and modified CeO_2_.

Figure 3 shows the XRD spectra of unmodified CeO_2_ and CeO_2_ modified with the inhibitor. The spectra endorsed the crystalline nature of CeO_2_ nanoparticles. Unmodified CeO_2_ nanoparticles showed high-intensity diffraction peaks at 2Ө = 28.49, 32.93, 47.44, and 56.14 at crystal planes of (111), (200), (220), and (311), respectively [24,25]. The diffraction peaks in the XRD spectra of CeO_2_ depicts the pure cubic structure, this result is by JCPDS No: 34-0394 of CeO_2_. The presence of the inhibitors in the CeO_2_ nanoparticles does not affect the crystalline nature of them. Hence, no effect in the diffraction peaks is seen.

### 3.2. FTIR Analysis of Unmodified, Modified CeO_2_ and Nanocomposite Coatings

**Figure 4:**
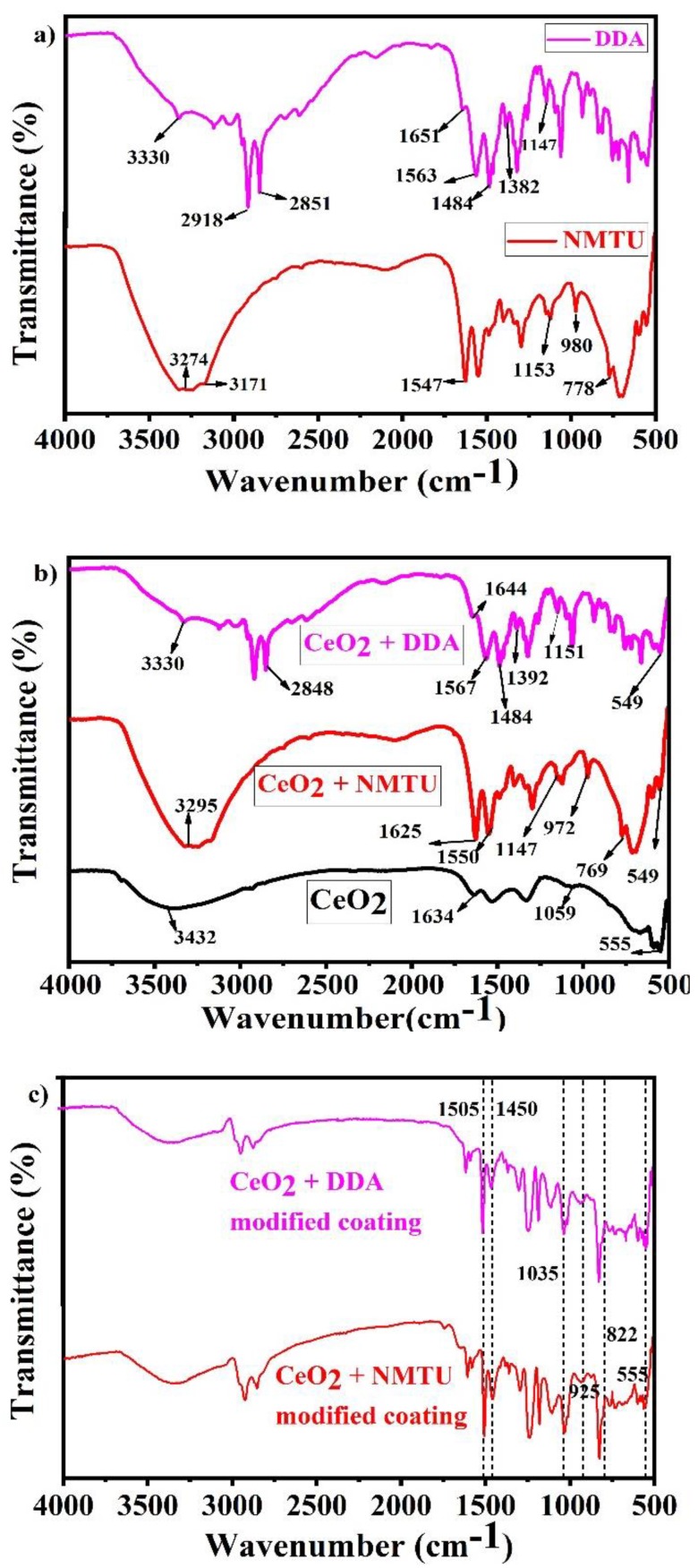
Fourier transform infrared (FTIR) spectra of (**a**) as-received inhibitors (NMTU and DDA), (**b**) unmodified and modified CeO_2_, and (**c**) nanocomposite smart coatings.

The FTIR of both pure NMTU and DDA, the unmodified and modified CeO_2_ nanoparticles with the two inhibitors, and the epoxy reinforced nanocomposite coatings are shown in Figure 4a–c, respectively.

As seen in Figure 4a, in case of NMTU, the peaks at 3274 and 3171 cm^−1^ are assigned to the –NH stretching and –NH_2_ symmetric vibrations, respectively. The peak at 1547 cm^−1^ corresponds to the thioamide group (–CSNH), which is due to the coupled vibrations of CS and CN stretching and NCN and CNC bending vibrations [26]. The band at 1153cm^−1^ represents the NH_2_ rocking vibrations. The methyl group (–CH_3_) is observed at 980 cm^−1^, while the sharp peak at 778 cm^−1^ corresponds to the C=S stretching vibrations. Whereas the peaks of NMTU in the FTIR spectrum, as shown in Figure 4b, are shifted from 1547 to 1550 cm^−1^, 1153 to 1147 cm^−1^, 980 to 972 cm^−1^, and 778 to 769 cm^−1^, after modification with CeO_2_. This shift is associated with the bonding involved in loading NMTU into CeO_2_ nanoparticles.

The FTIR spectra of DDA (Figure 4a) showed –NH_2_ deformation and –CN stretching vibrations band at 1651 and 1563 cm^−1^, respectively. The –CH_3_ deformation band is observed at 1484 cm^−1^ [27]. The peak shift seen in the DDA loaded CeO_2_ from 1651 to 1644 cm^−1^ (Figure 4b) can be assigned to an interaction between CeO_2_ and DDA. The band positioned at 2918 and 2851 cm^−1^ are assigned to the –CH_2_ symmetric and asymmetric stretching vibrations associated with DDA [28]. The peak at 3330 cm^−1^ represents the –NH_2_ stretching vibrations overlapping the –OH stretching vibrations [29].

As seen in Figure 4b, the FTIR spectrum of the unmodified CeO_2_ revealed the presence of some peaks positioned at 555, 1059, 1326, 1634, and 3423 cm^−1^. The peak at 3432 cm^−1^ corresponds to the O-H stretching vibrations, which are probably due to the adsorbed water on the surface of CeO_2_ nanoparticles [30]. The peak at 1634 cm^−1^ is associated with H_2_O (H-O-H bending frequency) [31]. The distinct peak at 555 cm^−1^ is attributed to the characteristic CeO_2_ (O-Ce-O) stretching vibrations. The comparison of the FTIR spectra of the unmodified and modified CeO_2_ nanoparticles with NMTU and DDA, as shown in Figure 4b, illustrated the successful loading of both inhibitors into the CeO_2_ nanoparticles.

In case of epoxy reinforced nanocomposite coatings (Figure 4c), the peaks at 822, 925, 1035, 1450, and 1505 cm^−1^ corresponds to oxirane, stretching of oxirane, stretching of C-O-C of ether, –CH_3_ deformation and C–C aromatic, respectively [32,33,34]. These confirm the successful inserting of the modified CeO_2_ nanoparticles into epoxy matrix, which leads to the development of nanocomposite coatings.

### 3.3. Thermogravimetric Analysis of Unmodified and Modified CeO_2_ Nanoparticles

**Figure 5:**
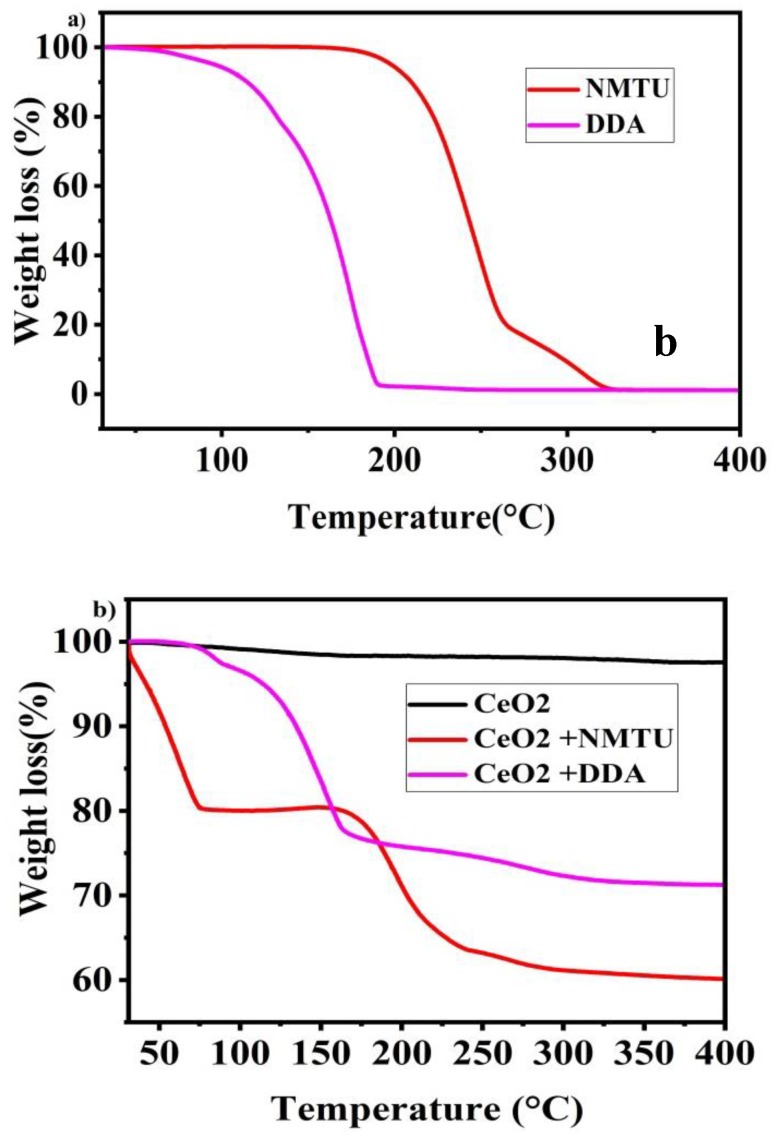

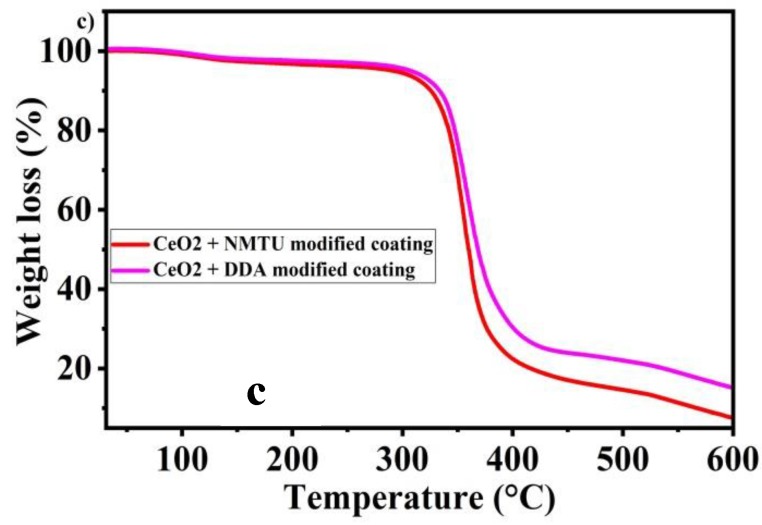
Thermal gravimetric analysis (TGA) curves of (**a**) as-received inhibitors (NMTU and DDA), (**b**) unmodified CeO_2_ and modified CeO_2_, and (**c**) Nanocomposite smart coating.

Thermogravimetric analysis of cerium dioxide nanoparticles, NMTU, DDA, CeO_2_/NMTU, CeO_2_/DDA, and epoxy reinforced nanocomposite coatings are shown in Figure 5. CeO_2_ shows good thermal stability, without any significant weight loss, as the decomposition of CeO_2_ could be seen beyond 400 °C (melting point = 3500 °C), as shown n Figure 5b. The slight weight loss of about 1–2% is probably due to the adsorbed water on the surface of cerium oxide.

Pure NMTU started to decompose at 166 °C until 320 °C, where no more of NMTU residue was left, as shown in Figure 5a. In the case of CeO_2_/NMTU (Figure 5b), weight loss is seen in three stages. The first stage of weight loss can be attributed to the loss of water content in the product. Then, the product was stable up to 157 °C. In the second stage of weight loss, the main degradation in weight is seen from 162 °C to 255 °C, which is due to the decomposition of the inhibitor that is on the surface of nanoparticles [35]. In the third stage, the weight loss is gradual, showing all of the NMTU has been decomposed. Moreover, the sudden weight loss at 165–400 °C indicates the absence of any absorbed water.

Pure DDA (Figure 5a) began to decompose from 104 °C to 192 °C until no more of it is left. Whereas the TGA analysis of CeO_2_/DDA (Figure 5b) starts with the initial loss of water content in the product of about 3%. In the second stage of weight loss from 97–164 °C, high weight loss, which is ascribed with the decomposition of DDA, is seen. After this, the weight loss is gradual until all DDA has been degraded at 327 °C (melting point = 250 °C).

By comparing the TGA diagrams of the modified CeO_2_ nanoparticles, it is perceived that inhibitors are burnt off at higher temperatures in the CeO_2_ nanoparticles loaded with inhibitors. This degradation is ascribed to the shield provided by the shell of CeO_2_ nano particles [23].

Based on the weight loss monitored by TGA measurements, we can also assume the amount of the inhibitor that loaded into the nanoparticles. Firstly, in case of CeO_2_/NMTU, we consider the weight of sample at 157 °C as G_1_ and at 400 °C as G_2,_ then, we subtract G_2_ from G_1_ (ΔG = G_1_ − G_2_), and lastly we divide ΔG by G_1_ to attain the ratio mass loss, rm =$\;\frac{\Delta G}{G_{1}}\times 100$ [23] due to NMTU. Therefore, for CeO_2_/NMTU, rm is about 25 wt.%. Similarly, in case of CeO_2_/DDA, we will consider G_1_ at 90.08 °C and G_2_ at 327 °C. Therefore, rm is 28.42 wt.%.

Thermal stability analysis of the synthesized nanocomposite coatings that are shown in Figure 5c do not show considerable weight loss until 350 °C, and a weight loss of 4–5% can be seen. However, after 350 °C there is substantial weight loss, (~80% to 85% when the temperature is increased from 350 °C to 500 °C). This weight loss can be ascribed to the breakdown of long chains in the epoxy matrix. After 500 °C, the weight loss is because of other additives in the coating [14,15].

### 3.4. pH-Responsive Behavior of Inhibitors

**Figure 6:**
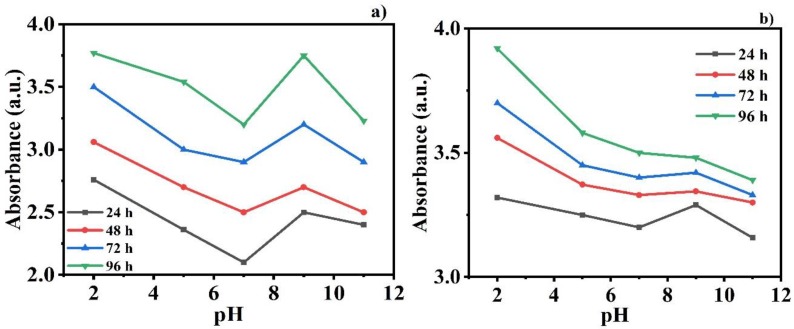
UV-vis analysis of (**a**) CeO_2_/NMTU and (**b**) CeO_2_/DDA after different time intervals of immersion in 3.5 wt.% NaCl solution at different pH values.

The self-releasing behavior of the inhibitors DDA and NMTU from CeO_2_ nanoparticles was recorded as a function of pH at various time intervals (24, 48, 72, and 96 h) using UV-vis spectroscopy. The modified CeO_2_ nanoparticles were incorporated in 3.5 wt.% NaCl solution at different pHs (2, 5, 7, 9, and 11). The change in the intensity of the absorbance peaks for the release of the DDA and NMTU inhibitor in different pH solutions at various time intervals can be seen in Figure 6a,b respectively. It is notable that the higher the value of the absorbance intensity, the greater the release amount of the inhibitor [36,37]. As we can see in Figure 6a, in the case of CeO_2_/NMTU, there are obvious changes in the absorbance at different pH values after 24 h immersion time. Likewise, the same trend can be observed for 48, 72, and 96 h immersion times. In addition, at each pH value, the absorbance increases as immersion time increases, indicating more release of the inhibitor, and confirming its time dependence, from CeO_2_ nanoparticles. Similar behavior is observed in the case of CeO_2_/DDA, as clarified in Figure 6b. However, the absorbance values in the case of DDA are larger than the corresponding ones of MNTU for the different immersion times at pH 7 (pH of 3.5 wt.% NaCl). This result coincided with the EIS results. It is further observed that the amount of inhibitor released from nanoparticles is higher at pH 2 and 9 compared to pH 5, 7, and 11. This difference in intensity at various pH and times showed that the self-release of the inhibitor is time dependent and pH sensitive. It is also notable that the release of the inhibitor is more favorable in the acidic medium, showing more sensitivity of inhibitors in the acidic medium compared to the alkaline one. This is due to the presence of the amino group in the inhibitors that works more efficiently at a lower pH [38].

### 3.5. Zeta Potential Measurements

The average zeta potential of the unmodified and modified CeO_2_ nanoparticles was studied at different pH values (2, 5, 7, 9, 11). The zeta potential was measured at different acidic and alkaline mediums, because as the steel substrate interacts with the electrolyte solution, the local pH at the scratch area starts to decrease, due to the hydrolysis of Fe^+^ [39]. As a result of this, the stability of the modified CeO_2_ nanoparticles increased, which caused the release of the inhibitor from the nanoparticles towards the defect site to get adsorbed on the metal/coating interface, thus protecting it from corrosion damages [27]. It is notable that the unmodified CeO_2_ nanoparticles have a positive zeta potential of 4.35 ± 1 mV at pH 2, and a negative zeta potential 0f −8.42 ± 1 mV at pH 11. The isoelectric point is observed at pH 5, as observed in the literature as well [40]. On the other hand, the loading of corrosion inhibitor NMTU and DDA in CeO_2_ nanoparticles caused the positive shift at pH 2 from 4.35 to 19.8 ± 1 mV and 22.4 ± 1 mV, respectively, and a negative shift from −8.42 mV to −9.06 ± 1 mV and −15.6 ± 1 mV, respectively. As both inhibitors have amine (-NH_2_) as their ionizing group, low pH facilitated the protonation of the amine group to NH_3_^+^, and high alkaline pH facilitated the deprotonation of amine group, thus attaining negative zeta potential [41]. This change in the zeta potential of the nanoparticles relative to the change in the pH makes the nanoparticles pH sensitive, thus releasing the inhibitor, as shown in Figure 6 as well.

### 3.6. Self-Healing of Inhibitor from Nanoparticles

**Figure 7:**
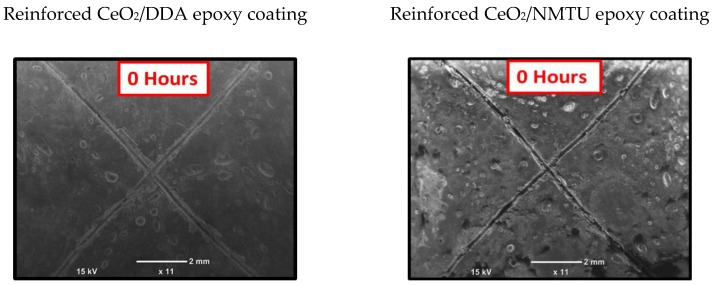

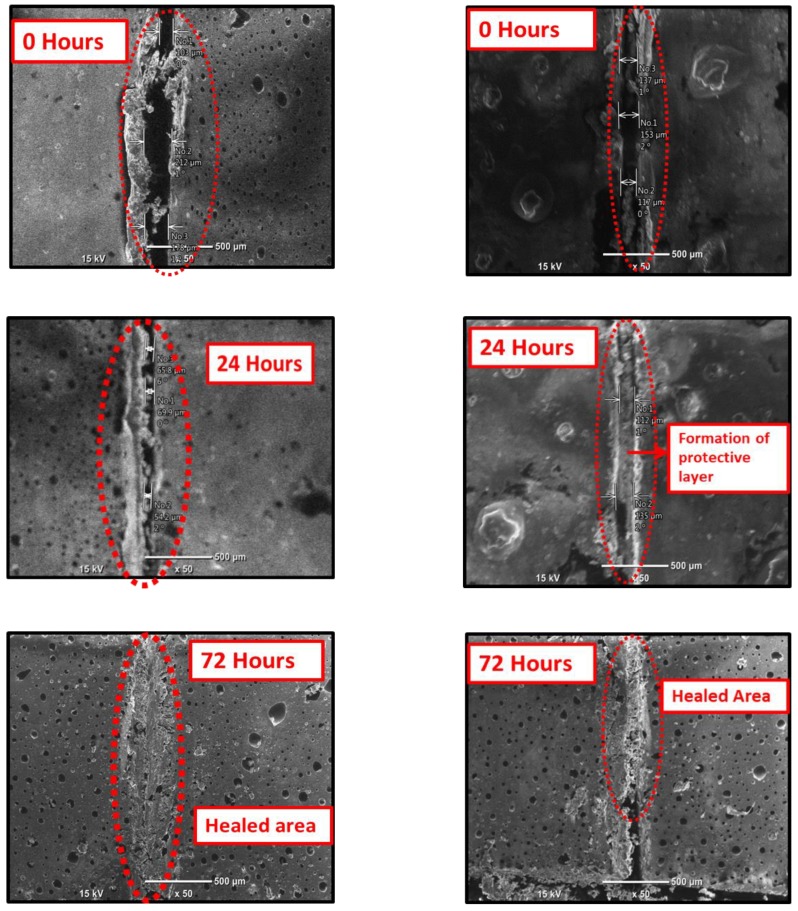
Scanning electron microscopy (SEM) images of the scratched reinforced CeO_2_/DDA (**left**) and CeO_2_/NMTU (**right**) epoxy coating at different time intervals (0, 24 and 72 h).

SEM was employed to assess the self-healing performance of the epoxy coatings that were reinforced with the modified CeO_2_ nanoparticles (CeO_2_/DDA and CeO_2_/NMTU) at different time intervals (0, 24, and 72 h), as presented in Figure 7. A controlled damage was introduced by making a scratch on the surface of the coated specimens reaching to the substrate using a sharp blade. The average scratch’s width was determined for each sample. As seen in Figure 7, there is a substantial decrease in the crack width over the passage of time for all the samples, indicating that the damaged area is healed in few hours. The possible healing mechanism of the protective coatings can also be seen in the graphical abstract. When an artificial defect was made, the steel starts to corrode, because of its interaction with the surrounding environment and the aggressive species such as water, oxygen, and chloride ions. This interaction leads to the change in the localized pH at the defect site, which stimulates the release of the inhibitor from the nanoparticles towards the defect, as described in the previous section. Then, the inhibitor adsorbs on the surface of the steel and forms a protective layer. In this way, the artificial defect that is created on the coated steel is covered, due to the mobility of the corrosion inhibitor towards the defect site, and thus the crack can be healed.

### 3.7. Corrosion Inhibition Behavior

**Figure 8:**
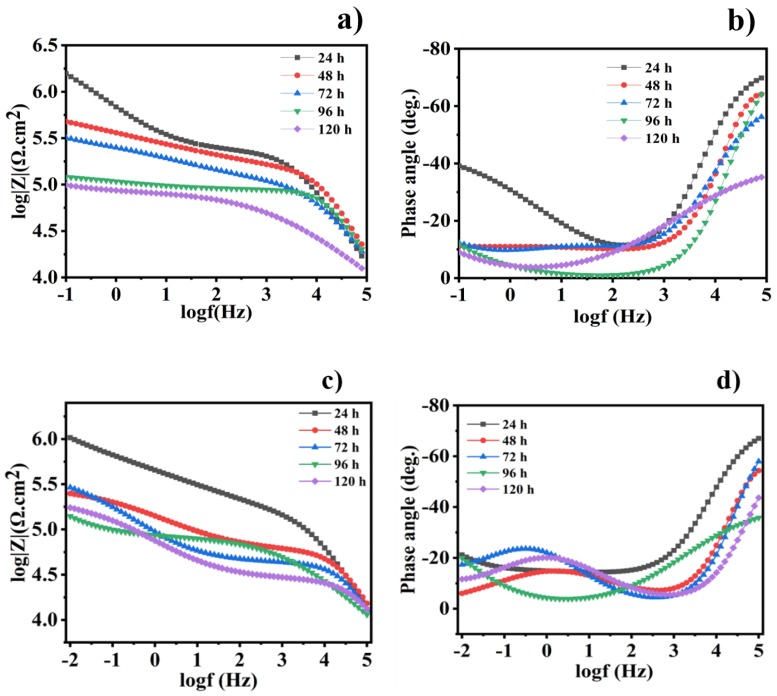
(**a**,**c**) Bode and (**b**,**d**) the corresponding phase angle plots for the scratched blank and epoxy/CeO_2_ coatings, respectively, after different immersion times in 3.5 wt.% NaCl at room temperature.

Electrochemical impedance spectroscopy (EIS) is considered to be the most powerful, non-destructive, and extensively useful electrochemical technique, and EIS measurements were performed to detect the corrosion protection of the coated steel samples at open circuit potential. Over various immersion times of 24, 48, 72, 96, and 120 h, the different scratched coatings (blank, epoxy/CeO_2_, epoxy/CeO_2_/DDA and epoxy/CeO_2_/NMTU) were immersed in 3.5 wt.% NaCl solutions at room temperature. Figure 8 shows bode and phase angle plots of the EIS spectra for the blank and epoxy/CeO_2_ coatings. The high impedance modulus at the low frequency |Z_0.1Hz_| value indicates the protective behavior of the coatings [42]. Figure 8a shows that the |Z_0.1Hz_| values for pure epoxy coatings decrease with the immersion time. In addition, as seen in Figure 8c, the |Z_0.1Hz_| values for the epoxy/CeO_2_ display the same trend as the CeO_2_-free epoxy coatings. However, the epoxy/CeO_2_ coating, along the different immersion times, attains higher corrosion resistance (|Z_0.1Hz_|) compared to that of the blank one (Figure 8c).

**Figure 9:**
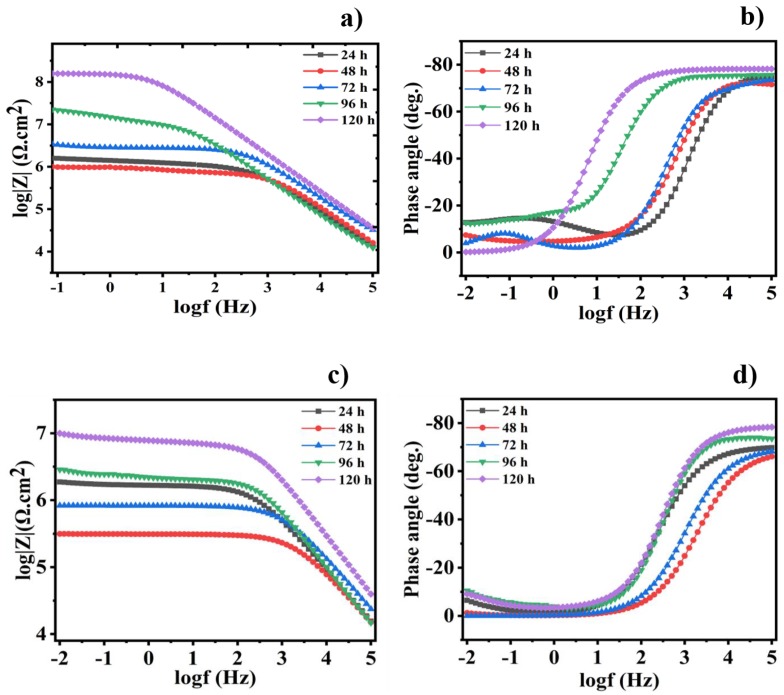
(**a**,**c**) Bode and (**b**,**d**) the corresponding phase angle plots for the scratched reinforced epoxy/CeO_2_/DDA and epoxy/CeO_2_/NMTU coatings, respectively, after different immersion times in 3.5 wt.% NaCl at room temperature.

Bode and phase angle plots for the epoxy/CeO_2_/DDA and epoxy/CeO_2_/NMTU coatings are shown in Figure 9. The |Z_0.1Hz_| values for the reinforced modified CeO_2_ coatings demonstrates the different trend compared to the CeO_2_-free and unmodified CeO_2_ ones. For example, the impedance modulus (|Z_0.1Hz_|) values for the reinforced modified CeO_2_ coatings decrease in the beginning of the immersion time and then, after 48 h, increase. Furthermore, it is worth mentioning that the addition of the inhibitors—either DDA or NMTU—into CeO_2_ nanoparticles causes a significant increase in the impedance modulus values through the different immersion times, as compared to the blank and the unmodified CeO_2_ specimens. The Nyquist plots of the different scratched coatings (blank, epoxy/CeO_2_, epoxy/CeO_2_/DDA and epoxy/CeO_2_/NMTU) are shown in Figure S2, which are in consistent with the corresponding bode plots.

**Figure 10:**
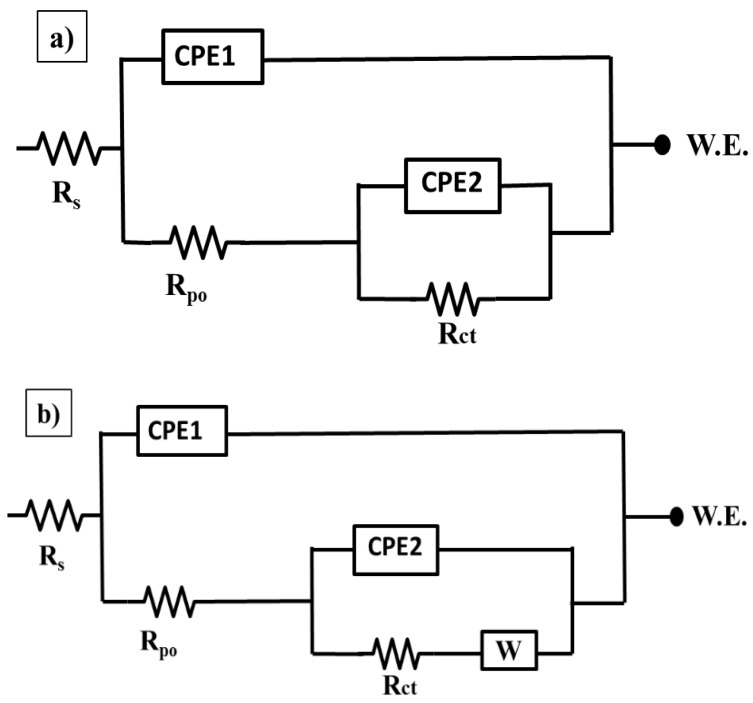
Equivalent circuits used to fit the impedance data for different epoxy coatings at different immersion times ((**a**): the first two days of immersion time; (**b**): a higher immersion time (72, 96, and 120 h)) in 3.5 wt.% NaCl solution at room temperature.

The impedance results for all coatings are fitted using the equivalent circuits that shown in Figure 10a,b and the electrochemical parameters of the fitted results are provided in Table 2. It is obvious that all the coatings have a two-time constants equivalent circuit, which is commonly used for the coated steel. The circuit consists of three different types of resistances named solution, pore, and charge transfer that are represented as *R*_s_, *R*_po_, and *R*_ct_, respectively. Additionally, it contains the constant phase elements (CPE_1_ and CPE_2_) and, after a certain immersion time, a Warburg impedance (*W)* is accounted. The high frequency time constant (*R*_po_/CPE_1_) is related to the coating’s properties. Whereas the low frequency time constant (*R*_ct_/CPE_2_) is referred to the electrochemical behavior at the interface between the substrate and the coating. The impedance of CPE is calculated using the following Equation (1) [34];

(1) $$Z_{CPE}=\frac{1}{Q^{o}\;{\left(jw\right)}^{\alpha }}$$

where *Q^o^* (s.Ω^−1^) equals to the (1/|Z|) at ω = 1 rad/s, ω is the angular frequency of the AC signal (1/rad) and *α* is the CPE exponent. As *α* reaches 1, the CPE shows ideal capacitor behavior [43]. Due to the non-homogeneity of all coatings, the CPE_1_ and CPE_2_ are used instead of the coating (C_c_) and double layer (C_dl_) capacitances, respectively, which are calculated using Equation (2) [44].

(2) $$C_{x}=\sqrt[{n}]{\frac{Q_{x}}{{R_{x}}^{\left(\alpha -1\right)}}}$$

where *α* is CPE exponent, *Q*_x_ is the CPE constant for the coating or the double layer, and *R*_x_ represents either the charge transfer resistance (*R*_ct_) or the pore resistance (*R*_po_).

As seen in Table 2, after 24 h of immersion, the *R*_ct_ and *R*_po_ values of the blank coating are 1.250 and 0.226 MΩ·cm^2^, which decreases after 120 h of immersion to become 0.249 and 0.029 MΩ·cm^2^, respectively. The decrease in the resistance values clearly depicts the deterioration of the protective ability, and an increase in the corrosion activity of the coatings. In addition, the CPE_1_ and CPE_2_ values increase with the increase of the immersion time, revealing that the blank coating is no longer offering good corrosion protection with the longer immersion time.

*R*_ct_ and *R*_po_ values of the epoxy/CeO_2_ coatings lose about 92% and 62.5% of their values after 120 h. of immersion and become 0.155 and 0.028 MΩ·cm^2^, respectively, which are still higher than those of the blank coating. This indicates that the epoxy/CeO_2_ coating has some protection behavior, due to the presence of CeO_2_ nanoparticles in the epoxy coating that reduce its porosity and contributes to its reinforcement [20]. Then, its protection ability decreases gradually with immersion time, because of the penetration of the electrolytes in the defect environment and the absence of the self-healing agent. In addition, it is demonstrated the inconstancy in the *R*_po_ values of the epoxy/CeO_2_ coating over the immersion time indicating the deterioration of the coating itself. As clarified in Table 2, and seen in Figure 8b,d, and Figure 9b,d, which have two relaxation times observed at high and low frequencies, the equivalent circuit that was employed for the blank and epoxy/CeO_2_ coatings, through all immersion times, is shown in Figure 10a.

The *R*_ct_ and *R*_po_ values of the reinforced modified coatings, after 120 h immersion time, are enhanced by 98- and 61-fold, respectively, in case of epoxy/CeO_2_/DDA and by 6.0- and 7.8-fold, respectively, for epoxy/CeO_2_/NMTU, compared to their values after 24 h immersion time. The enhancement of the resistance values and, consequently, the protection ability of the epoxy coatings in the presence of the modified CeO_2_, is related to the good compatibility of the CeO_2_ nanoparticles with the epoxy, and the good inhibition property of the inhibitors (DDA and NMTU) [45]. This is in addition to their self-healing ability that is attributed to the release and adsorption of the inhibitors on the defect site of the steel substrate. At the same time, the CPE_1_ and CPE_2_ values of both epoxy/CeO_2_/DDA and epoxy/CeO_2_/NMTU coatings have the lowest values with a longer immersion time. Furthermore, the CPE_1_ and CPE_2_ values of the epoxy/CeO_2_/DDA are less than those of the epoxy/CeO_2_/NMTU. Moreover, Figure 9b,d indicate that, after 120 h immersion time, the phase angles at higher frequencies of the epoxy/CeO_2_/DDA and epoxy/CeO_2_/NMTU coatings are close to −80° and −70°, respectively, illustrating the capacitance behavior and the protective ability of both modified coatings. Obviously, the protection behavior of the reinforced modified CeO_2_ epoxy coatings follows the order of epoxy/CeO_2_/DDA > epoxy/CeO_2_/NMTU.

Table 2 shows that the epoxy/CeO_2_/DDA coating have a higher *IE*% (99.8%), after 120 h of immersion in a 3.5 wt.% solution, than that of epoxy/CeO_2_/NMTU (95.7%). The corrosion inhibition efficiency (*IE*%) can be calculated as follows [44].

(3) $$IE\%\;=\;(1-R_{ct1}/R_{ct2})\times 100$$

where *R*_ct1_ and *R*_ct2_ are the charge transfer resistances of the blank and coated specimens, respectively. The protection efficiency (*IE*) of the coated specimens at different immersion times is calculated related to *R*_ct_ value of the blank one after 24 h immersion time.

As seen in Figure 9a,c and Table 2, it is noted that, after 48 h of immersion time, there is a decrease in the *R*_ct_ values from 1.7 to 1.4 MΩ·cm^2^ in case of epoxy/CeO_2_/DDA and from 1.1 to 0.132 MΩ·cm^2^ in the case of epoxy/CeO_2_/NMTU coatings. This could be attributed to the presence of some pores in the coatings that allow the uptake of electrolyte solution and the formation of conductive paths. In addition, the uptake of the electrolyte solution occurs by the diffusion of the electrolyte through the scratched area. The epoxy/CeO_2_/DDA and epoxy/CeO_2_/NMTU coatings, during the first two days of immersion time, were fitted by the equivalent circuit of Figure 10a.

The equivalent circuit in Figure 10b is used to fit the epoxy/CeO_2_/DDA and epoxy/CeO_2_/NMTU coatings at a higher immersion time (72, 96, and 120 h). Warburg impedance (*W*) is introduced to show that the inhibitors have affected the electrode process [46]. For example, after 72 h immersion time, the *R*_ct_ values of both coatings are gradually increased until 120 h. This is due to the gradual release of the inhibitors that have an amine group, which acts as a hard base in both the DDA and NMTU [47].

The amine group forms a protective layer, which prevents the free movement of the corrosive species or ions and, subsequently, preventing it from reaching the scratched area [48]. When the scratched area is in contact with the NaCl solution, the iron oxide, which is acidic, will cover this area [49]. Due to the hydrolysis of Fe^+^ cations of the steel substrate, the pH at the scratched area starts to decrease, and stimulate the inhibitors to be released from the CeO_2_ nanoparticles and form the protective layer. This is in agreement with the previous studies [38], which proved that amine based inhibitors like DDA and NMTU work more efficiently in an acidic medium, leading to the formation of a protective layer and providing self-healing functionality.

**Figure 11:**
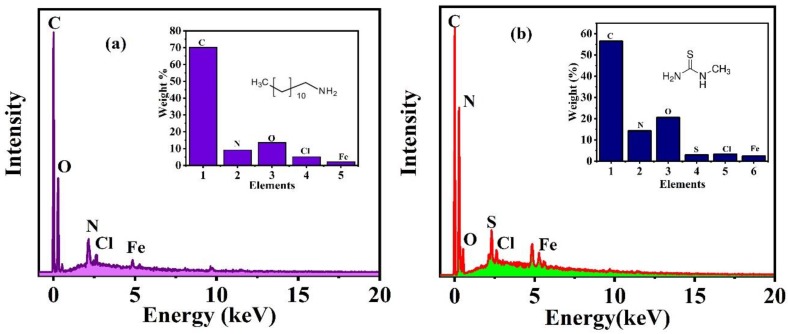
EDX analysis of the protective film formed on the scratched area of the reinforced epoxy coated samples with (**a**) CeO_2_/DDA (**b**) CeO_2_/NMTU.

To confirm the formation of the protective film, the EDX, as shown in Figure 11, is done at the scratched area of the substrate that was exposed to the NaCl solution for 96 h. The weight % of the elements observed through EDX is shown in inset. The appearance of O, N, and C, which are the main components of the DDA inhibitor, at the defect site of the reinforced epoxy coated sample with CeO_2_/DDA, which indicates the presence of this inhibitor and its good release, as shown in Figure 11a. Similarly, the existence of C, N, and S, as shown in Figure 11b, in the scratched area of the reinforced epoxy coated sample with CeO_2_/NMTU, after immersing in the NaCl solution for 96 h, which emphasizes the release ability of the NMTU. The results are in consistent with our previous results shown in Figure 7 and Figure 9.

## 4. Conclusions

In this research, two different organic inhibitors, DDA and NMTU, are loaded CeO_2_ nanoparticles and incorporated in the epoxy coating for corrosion protection of steel in 3.5 wt.% NaCl solution. FTIR analysis confirmed the successfully loading of the inhibitors in CeO_2_ nanoparticles. TGA measurements clarified the loading amount of inhibitors by weight loss measurements. UV-vis spectroscopy and zeta potential measurements validated the time dependent and pH responsive release of inhibitors form nanoparticles. SEM analysis investigated the release of the inhibitors and formation of the protective layer at the scratched area. The corrosion resistance of the scratched reinforced epoxy coatings with CeO_2_/DDA and CeO_2_/NMTU was evaluated using EIS. The results illustrated an exceptional protection efficiency reaching 99.8% and 95.7% for the modified epoxy coating with DDA and NMTU, respectively, after 5 days of immersion in the chloride solution. The enhanced inhibition properties of the modified epoxy coatings, as compared to the blank epoxy coatings, are ascribed to the release of the inhibitor and its self-healing functionalities that make them attractive for use in the oil and gas industry.

## Figures and Tables

**Table 1:** Elemental Analysis of unloaded and loaded CeO_2_ nanoparticles.

Samples	Element Present	wt.%	at.%
Unloaded CeO_2_	Ce, O	Ce = 75.96, O = 16.22	Ce = 24.68, O = 46.15
CeO_2_/DDA (C_43_H_53_NO_14_)	Ce, O, C, N	Ce = 62.84, O = 14.72, C = 21.60, N = 0.48	Ce = 13.95, O = 28.63, C = 55.97, N = 1.06
CeO_2_/NMTU (NH_2_CSNHCH_3_)	Ce, O, C, N	Ce = 62.67, O = 14.79, C = 22.22, N = 0.23	Ce = 13.84, O = 28.60, C = 57.23, N = 0.34

**Table 2:** The obtained fitted electrochemical values of the electrochemical impedance spectroscopy (EIS) data for the different coated specimens immersed in 3.5 wt.% NaCl for different immersion times at room temperature.

Coatings	Time (h)	*R*_po_ (MΩ·cm^2^)	CPE_1_ (F·cm^−2^·S^n−1^)	*R*_ct_ (MΩ·cm^2^)	CPE_2_ (F·cm^−2^·S^n−1^)	*W* (MΩ·cm^2^·S^−1/2^)	*IE (%)*
blank epoxy	24	0.226	1.608 × 10^−7^	1.250	1.522 × 10^−10^	-	-
	48	0.106	7.701 × 10^−6^	1.172	4.259 × 10^−9^		
	72	0.104	7.34 × 10^−6^	0.714	2.605 × 10^−9^		
	96	0.084	2.908 × 10^−6^	0.549	1.488 × 10^−9^		
	120	0.029	1.08 × 10^−6^	0.249	1.206 × 10^−9^		
Epoxy/CeO_2_	24	0.075	1.459 × 10^−6^	1.947	9.518 × 10^−10^	-	84.9
	48	0.043	6.613 × 10^−6^	0.266	1.048 × 10^−9^		-
	72	0.055	3.710 × 10^−6^	0.235	1.280 × 10−9		
	96	0.022	3.532 × 10^−6^	0.166	1.408 × 10^−9^		
	120	0.028	6.562 × 10^−6^	0.155	1.994 × 10^−9^		
Epoxy/CeO_2_/DDA	24	0.705	4.794 × 10^−8^	1.708	1.014 × 10^−9^	-	82.8
	48	0.313	8.047 × 10^−7^	1.408	2.175 × 10^−9^	-	-
	72	0.966	1.628 × 10^−9^	2.238	5.937 × 10^−10^	2.83	-
	96	10.54	6.077 × 10^−10^	9.123	2.289 × 10^−10^	11.0	-
	120	42.77	8.101 × 10^−11^	167.3	1.081 × 10^−10^	47.5	99.8
Epoxy/CeO_2_/NMTU	24	0.205	2.907 × 10^−7^	1.125	1.885 × 10^−9^	-	73.9
	48	0.013	3.969 × 10^−7^	0.132	2.461 × 10^−9^	-	-
	72	0.839	1.625 × 10^−8^	0.695	6.962 × 10^−10^	1.70	-
	96	1.076	3.627 × 10^−9^	1.831	4.471 × 10^−10^	10.5	-
	120	1.614	1.233 × 10^−9^	6.892	1.145 × 10^−10^	13.5	95.7

## Data Availability

The raw/processed data required to reproduce these findings cannot be shared at this time due to legal or ethical reasons.

## Supplementary Materials

The following are available online at https://www.mdpi.com/2079-4991/10/4/791/s1, Figure S1: EDS analysis of (a) unmodified CeO_2_ nanoparticles (b) modified CeO_2_/DDA (c) CeO_2_/NMTU, Figure S2: Nyquist plots of (a) blank epoxy, (b) epoxy/CeO_2_, (c) epoxy/CeO_2_/DDA and (d) epoxy/CeO_2_/NMTU coatings.

## References

[1] Revie R.W., Uhlig H.H. (2008). Corrosion and Corrosion Control: An Introduction to Corrosion Science and Engineering.

[2] Montemor M.F., Vicente C., Técnico I.S. (2018). Functional Self-Healing Coatings: A New Trend in Corrosion Protection by Organic Coatings.

[3] Philip A., Schweitzer P.E. (2005). Paint and Coatings: Applications and Corrosion Resistance.

[4] Nawaz M., Habib S., Khan A., Shakoor R.A., Kahraman R. (2020). Cellulose microfibers (CMFs) as a smart carrier for autonomous self-healing in epoxy coatings. New J. Chem..

[5] Habib S., Khan A., Nawaz M., Sliem M.H., Shakoor R.A., Kahraman R., Abdullah A.M., Zekri A. (2019). Self-healing performance of multifunctional polymeric smart coatings. Polymers (Basel).

[6] Tang L., Whalen J., Schutte G., Weder C. (2009). Stimuli-responsive epoxy coatings. ACS Appl. Mater. Interfaces.

[7] Cotting F., Aoki I.V. (2015). Smart protection provided by epoxy clear coating doped with polystyrene microcapsules containing silanol and Ce (III) ions as corrosion inhibitors. Surf. Coat. Technol..

[8] Behpour M., Ghoreishi S.M., Khayatkashani M., Soltani N. (2011). The effect of two oleo-gum resin exudate from Ferula assa-foetida and Dorema ammoniacum on mild steel corrosion in acidic media. Corros. Sci..

[9] Dehri I., Erbil M. (2004). Organic sulphur-containing compounds as corrosion inhibitors for mild steel in acidic media: Correlation between inhibition efficiency and chemical structure. Appl. Surf. Sci..

[10] Zahidah K.A., Kakooei S., Ismail M.C., Bothi Raja P. (2017). Halloysite nanotubes as nanocontainer for smart coating application: A review. Prog. Org. Coat..

[11] Lvov Y.M., Shchukin D.G., Mohwald H., Price R.R. (2008). Halloysite clay nanotubes for controlled release of protective agents. ACS Nano.

[12] Poornima Vijayan P., Al-Maadeed M.A.S.A. (2016). TiO_2_ nanotubes and mesoporous silica as containers in self-healing epoxy coatings. Sci. Rep..

[13] Nguyen T.A., Pham T.L., Dinh TM T., Thai H., Shi X. (2017). Application of Nano-SiO_2_ and Nano-Fe_2_O_3_ for Protection of Steel Rebar in Chloride Contaminated Concrete: Epoxy Nanocomposite Coatings and Nano-Modified Mortars. J. Nanosci. Nanotechnol..

[14] Van Soestbergen M., Baukh V., Erich S.J.F., Huinink H.P., Adan O.C.G. (2014). Release of cerium dibutylphosphate corrosion inhibitors from highly filled epoxy coating systems. Prog. Org. Coat..

[15] Thuy N., Thi T., Hang X., Nicolay A., Paint Y. (2016). Progress in Organic Coatings Corrosion protection of carbon steel by solvent free epoxy coating containing hydrotalcites intercalated with different organic corrosion inhibitors. Prog. Org. Coat..

[16] Sastri V.S., Perumareddi J.R. (1997). Molecular Orbital Theoretical Studies of Some Organic Corrosion Inhibitors. Corrosion.

[17] Lamaka S.V., Zheludkevich M.L., Yasakau K.A., Montemor M.F., Ferreira M.G.S. (2007). High effective organic corrosion inhibitors for 2024 aluminium alloy. Electrochim. Acta.

[18] Khramov A.N., Voevodin N.N., Balbyshev V.N., Mantz R.A. (2005). Sol—Gel-derived corrosion-protective coatings with controllable release of incorporated organic corrosion inhibitors. Thin Solid Films.

[19] Fayyad E.M., Almaadeed M.A., Jones A., Abdullah A.M. (2014). Evaluation techniques for the corrosion resistance of self-healing coatings. Int. J. Electrochem. Sci..

[20] Liu X., Gu C., Wen Z., Hou B. (2018). Improvement of active corrosion protection of carbon steel by water-based epoxy coating with smart CeO_2_ nanocontainers. Prog. Org. Coat..

[21] Zand R.Z., Flexer V., De Keersmaecker M., Verbeken K., Adriaens A. (2015). Effects of activated ceria and zirconia nanoparticles on the protective behaviour of silane coatings in chloride solutions. Int. J. Electrochem. Sci..

[22] Pirhady Tavandashti N., Ghorbani M., Shojaei A., Gonzalez-Garcia Y., Terryn H., Mol J.M.C. (2016). pH responsive Ce(III) loaded polyaniline nanofibers for self-healing corrosion protection of AA2024-T3. Prog. Org. Coat..

[23] Mekeridis E., Kartsonakis I., Pappas G. (2011). Release studies of corrosion inhibitors from Cerium Titanium Oxide Release studies of corrosion inhibitors from cerium titanium oxide nanocontainers. J. Nanopart. Res..

[24] Chelliah M., Rayappan J.B.B., Krishnan U. (2012). Synthesis and Characterization of Cerium Oxide Nanoparticles by Hydroxide Mediated Approach. J. Appl. Sci..

[25] Renu G., Divya Rani V.V., Nair S.V., Subramanian K.R.V., Lakshmanan V.K. (2012). Development of cerium oxide nanoparticles and its cytotoxicity in prostate cancer cells. Adv. Sci. Lett..

[26] Mido Y., Kitagawa I., Hashimoto M., Matsuura H. (1999). Vibrational spectra and normal coordinate analysis of N-methylthiourea and three deuterated analogues. Spectrochim. Acta Part A Mol. Biomol. Spectrosc..

[27] Ubaid F., Radwan A.B., Naeem N., Shakoor R.A., Ahmad Z., Montemor M.F., Kahraman R., Abdullah A.M., Soliman A. (2019). Multifunctional self-healing polymeric nanocomposite coatings for corrosion inhibition of steel. Surf. Coat. Technol..

[28] Iacob M., Cazacu M., Racles C., Ignat M., Cozan V., Sacarescu L., Timpu D., Kajňaková M., Botko M., Feher A. (2014). Iron-chromium oxide nanoparticles self-assembling into smectic mesophases. RSC Adv..

[29] de Menezes B.R.C., Ferreira F.V., Silva B.C., Simonetti E.A.N., Bastos T.M., Cividanes L.S., Thim G.P. (2018). Effects of octadecylamine functionalization of carbon nanotubes on dispersion, polarity, and mechanical properties of CNT/HDPE nanocomposites. J. Mater. Sci..

[30] Ali M.M., Mahdi H.S., Parveen A., Azam A. (2018). Optical properties of cerium oxide (CeO_2_) nanoparticles synthesized by hydroxide mediated method. AIP Conf. Proc..

[31] Wu N.C., Shi E.W., Zheng Y.Q., Li W.J. (2002). Effect of pH of medium on hydrothermal synthesis of nanocrystalline cerium(IV) oxide powders. J. Am. Ceram. Soc..

[32] Maiorana A., Spinella S., Gross R.A. (2015). Bio-Based Alternative to the Diglycidyl Ether of Bisphenol A with Controlled Materials Properties. Biomacromolecules.

[33] Carja I.-D., Serbezeanu D., Vlad-Bubulac T., Hamciuc C., Coroaba A., Lisa G., López C.G., Soriano M.F., Pérez V.F., Romero Sánchez M.D. (2014). A straightforward, eco-friendly and cost-effective approach towards flame retardant epoxy resins. J. Mater. Chem. A.

[34] González M.G., Cabanelas J.C., Baselga J. (2012). Applications of FTIR on Epoxy Resins - Identification, Monitoring the Curing Process, Phase Separation and Water Uptake. Infrared Spectrosc. Mater. Sci. Eng. Technol..

[35] Mariappan M., Madhurambal G., Ravindran B., Mojumdar S.C. (2011). Thermal, FTIR and microhardness studies of bisthiourea-urea single crystal. J. Therm. Anal. Calorim..

[36] Njoku D.I., Cui M., Xiao H., Shang B., Li Y. (2017). Understanding the anticorrosive protective mechanisms of modified epoxy coatings with improved barrier, active and self-healing functionalities: EIS and spectroscopic techniques. Sci. Rep..

[37] Feng Y., Cheng Y.F. (2017). An intelligent coating doped with inhibitor-encapsulated nanocontainers for corrosion protection of pipeline steel. Chem. Eng. J..

[38] Nawaz M., Yusuf N., Habib S., Shakoor R.A., Ubaid F., Ahmad Z., Kahraman R., Mansour S., Gao W., Nawaz M. (2019). Development and Properties of Polymeric Nanocomposite Coatings. Polymers (Basel).

[39] Qi Y., Li X., He Y., Zhang D., Ding J. (2019). Mechanism of Acceleration of Iron Corrosion by a Polylactide Coating. ACS Appl. Mater. Interfaces.

[40] Dahle J.T., Livi K., Arai Y. (2015). Effects of pH and phosphate on CeO_2_ nanoparticle dissolution. Chemosphere.

[41] Li Q., Zhang H., Tu Z., Yu J., Xiong C., Pan M. (2012). Impregnation of amine-tailored titanate nanotubes in polymer electrolyte membranes. J. Memb. Sci..

[42] Fu J., Chen T., Wang M., Yang N., Li S., Wang Y., Liu X. (2013). Acid and Alkaline Dual Stimuli-Responsive Mechanized Hollow Mesoporous Silica Nanoparticles as Smart Nanocontainers for Intelligent Anticorrosion Coatings. ACS Nano.

[43] Bahgat Radwan A., Ali K., Shakoor R.A., Mohammed H., Alsalama T., Kahraman R., Yusuf M.M., Abdullah A.M., Fatima Montemor M., Helal M. (2018). Properties enhancement of Ni-P electrodeposited coatings by the incorporation of nanoscale Y_2_O_3_ particles. Appl. Surf. Sci..

[44] Jlassi K., Radwan A.B., Sadasivuni K.K., Mrlik M., Abdullah A.M., Chehimi M.M., Krupa I. (2018). Anti-corrosive and oil sensitive coatings based on epoxy/polyaniline/magnetite-clay composites through diazonium interfacial chemistry. Sci. Rep..

[45] Verma C., Quraishi M.A., Singh A. (2015). 2-Amino-5-nitro-4,6-diarylcyclohex-1-ene-1,3,3-tricarbonitriles as new and effective corrosion inhibitors for mild steel in 1 M HCl: Experimental and theoretical studies. J. Mol. Liq..

[46] Cheng Y.L., Zhang Z., Cao F.H., Li J.F., Zhang J.Q., Wang J.M., Cao C.N. (2004). A study of the corrosion of aluminum alloy 2024-T3 under thin electrolyte layers. Corros. Sci..

[47] Welle A., Liao J.D., Kaiser K., Grunze M., Mäder U., Blank N. (1997). Interactions of N,N′-dimethylaminoethanol with steel surfaces in alkaline and chlorine containing solutions. Appl. Surf. Sci..

[48] Fekry A.M., Mohamed R.R. (2010). Acetyl thiourea chitosan as an eco-friendly inhibitor for mild steel in sulphuric acid medium. Electrochim. Acta.

[49] Zhou X., Yang H.-Y., Wang F.-H. (2011). Corrosion inhibition by sorbitol/diethylenetriamine condensation product for carbon steel in 3.5% NaCl saturated Ca(OH)_2_ solution. Acta Phys. Chim. Sin..

