# Supplementary material for: Cerium Dioxide Nanoparticles as Smart Carriers for Self-Healing Coatings
Source: Nanomaterials (Basel). 2020 Apr 20;10(4):791. doi: 10.3390/nano10040791 (PMC7221694; doi:10.3390/nano10040791)
Supplement: Supplementary file 1 [file nanomaterials-10-00791-s001.pdf]

Supplementary Information

Cerium dioxide nanoparticles as smart carriers for self-healing coatings

Sehrish Habib[1], Eman Fayyad[1,a], Muddasir Nawaz[1], Adnan Khan[1], R. A. Shakoor [1*], Ramazan Kahraman[2], Aboubakr Abdullah[1]

[1]Center for Advanced Materials (CAM), Qatar University, 2713 Doha, Qatar.

[2]Department of Chemical Engineering, Qatar University, 2713 Doha, Qatar.

[*] Corresponding Authors: shakoor@qu.edu.qa, Tel: +974–44036867, 974-6612-5107
[a] Permanent address: Physical Chemistry Department, National Research Centre, P.O. Box 12622, Dokki, Giza, Egypt.

Supplementary Information 1

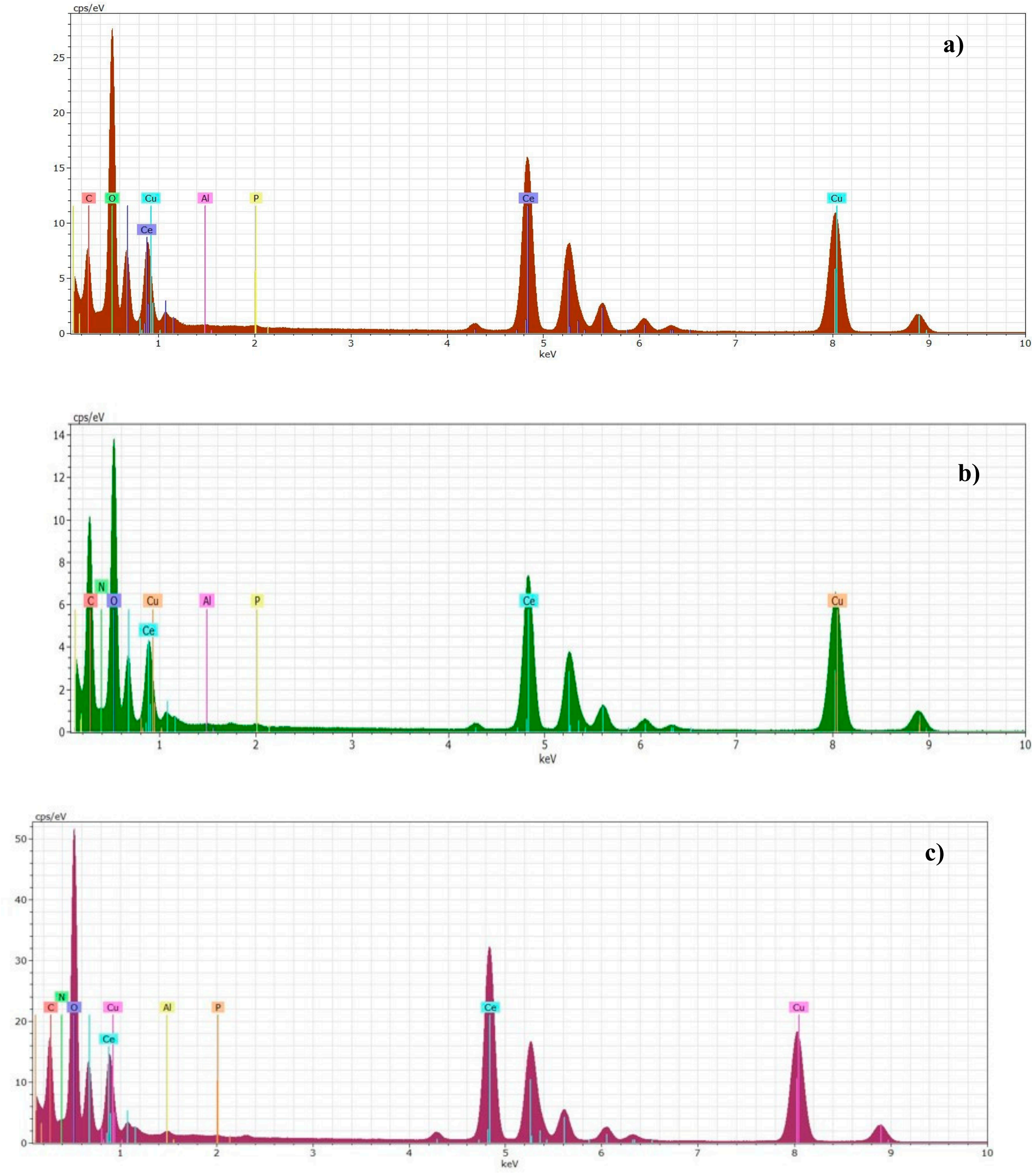

Figure S1: EDS analysis of a) unmodified CeO_2 nanoparticles b) modified CeO_2/DDA c) CeO_2/NMTU

Supplementary Information 2

Nyquist plot

The Nyquist plots, which is measured at open circuit potential, for blank epoxy, epoxy/CeO_2, epoxy/CeO_2/DDA and epoxy/CeO_2/NMTU coatings in 3.5 wt % NaCl are presented in Figure S2. All curves appear in a semicircular in the analyzed frequency range of 100 KHz to 0.01 Hz. However, the epoxy/CeO_2/DDA and epoxy/CeO_2/NMTU coatings curves after 72h immersion time were a semicircle with a straight line at lower frequencies, which may be indicative of electrolyte penetration within the coating. It is known that the smaller the Nyquist semicircle diameter, the smaller the charge transfer resistance through the metal/electrolyte solution interface, and consequently, the higher the corrosion rate is. It can be observed that the diameter of semicircle decreases with time as the charge transfer resistance (R_{ct}) value decreases for the blank epoxy coating. The R_{ct} value for blank epoxy decreased from 1.6 MΩ.cm^2 to 29 KΩ.cm^2 due to corrosion activity. The electrochemical behavior for epoxy/CeO_2 is similar to the blank epoxy where the value of R_{ct} decreased from 600 KΩ.cm^2 to 200 KΩ.cm^2 due to the absence of the inhibitor. The diameter of the Nyquist plot for epoxy/CeO_2/DDA coating shows an increase in R_{ct} value with time due to better corrosion inhibition. The increase in R_{ct} value is due to the release of DDA from the CeO_2 nanoparticles that helps to develop the protective layer on the steel substrate. Furthermore, it is observed that the R_{ct} value of the epoxy/CeO_2/DDA coating increased from ~2 MΩ.cm^2 after 24 h to 160 MΩ.cm^2 after 96 h immersion in the electrolyte. This is due to the efficient corrosion inhibition of the inhibitor. For epoxy/CeO_2/NMTU coatings, the initially R_{ct} value slightly decreased from 2 MΩ.cm^2 to 500 KΩ.cm^2 after 48 h of immersion. Then, once the protective layer developed on the steel substrate due to the release of NMTU from CeO_2, the R_{ct} values increased reaching to 10 MΩ.cm^2 after 120 h of immersion. The Nyquist plots of the different coatings are in consistent with the corresponding bode plots.

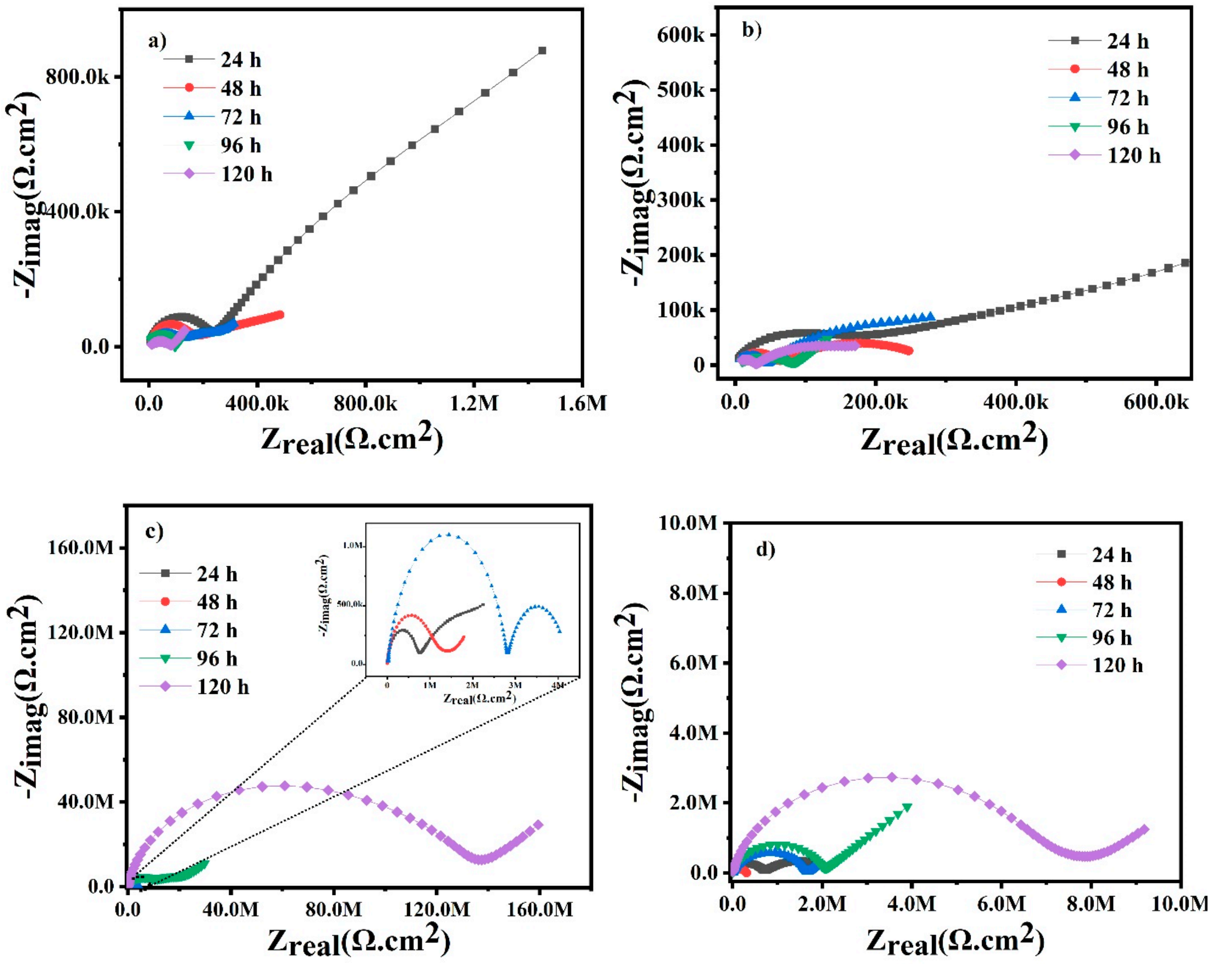

Figure S2: Nyquist plots of a) blank epoxy, b) epoxy/CeO_2, c) epoxy/CeO_2/DDA and d) epoxy/CeO_2/NMTU coatings.
